# Comprehensive analysis of little leaf disease incidence and resistance in eggplant

**DOI:** 10.1186/s12870-024-05257-4

**Published:** 2024-06-18

**Authors:** Muthusamy Karthikeyan, Gawande Priya Yogiraj, Thiyagarajan Elaiyabharathi, Bonipas Antony John Jesu, Iruthayasamy Johnson, Shajith Basha Jaffer, Shanmuga Priya Dhanabalan, Narayanan Manikanda Boopathi, Subbaiyan Marimuthu, Hamid Shobeiri Nejad, Dante L. Adorada, Sambasivam Periyannan

**Affiliations:** 1 Department of Plant Pathology, Tamil Nadu Agricultural University, Coimbatore, 641003 Tamil Nadu India; 2 Centre for Crop Health, University of Southern Queensland, Toowoomba, QLD 4350 Australia; 3 Department of Agricultural Entomology, Tamil Nadu Agricultural University, Coimbatore, 641003 Tamil Nadu India; 4 Department of Plant Breeding and Genetics, Tamil Nadu Agricultural University, Coimbatore, 641003 Tamil Nadu India; 5 Department of Biotechnology, Tamil Nadu Agricultural University, Coimbatore, 641003 Tamil Nadu India; 6 National Pulses Research Centre, Tamil Nadu Agricultural University, Vamban, 622303 Tamil Nadu India; 7 Centre for Applied Climate Sciences, University of Southern Queensland, Toowoomba, QLD 4350 Australia; 8 School of Mathematics, Physics and Computing, University of Southern Queensland, Toowoomba, QLD 4350 Australia; 9 School of Agriculture and Environmental Science, University of Southern Queensland, Toowoomba, QLD 4350 Australia

**Keywords:** Eggplant, Little leaf, Phytoplasma, Leafhopper-*Hishimonas phycitis*, Resistant genotypes

## Abstract

**Background:**

Little leaf disease caused by phytoplasma infection is a significant threat to eggplant (also known as brinjal) cultivation in India. This study focused on the molecular characterisation of the phytoplasma strains and insect vectors responsible for its transmission and screening of brinjal germplasm for resistance to little leaf disease.

**Results:**

Surveys conducted across districts in the Tamil Nadu state of India during 2021–2022 showed a higher incidence of phytoplasma during the Zaid (March to June), followed by Kharif (June to November) and Rabi (November to March) seasons with mean incidence ranging from 22 to 27%. As the name indicates, phytoplasma infection results in little leaf (reduction in leaf size), excessive growth of axillary shoots, virescence, phyllody, stunted growth, leaf chlorosis and witches’ broom symptoms. PCR amplification with phytoplasma-specific primers confirmed the presence of this pathogen in all symptomatic brinjal plants and in *Hishimonus phycitis* (leafhopper), providing valuable insights into the role of leafhoppers in disease transmission. BLAST search and phylogenetic analysis revealed the phytoplasma strain as “Candidatus Phytoplasma trifolii”. Insect population and disease dynamics are highly influenced by environmental factors such as temperature, relative humidity and rainfall. Further, the evaluation of 22 eggplant accessions revealed immune to highly susceptible responses where over 50% of the entries were highly susceptible. Finally, additive main effect and multiplicative interaction (AMMI) and won-where biplot analyses identified G18 as a best-performing accession for little leaf resistance due to its consistent responses across multiple environments.

**Conclusions:**

This research contributes essential information on little leaf incidence, symptoms, transmission and resistance profiles of different brinjal genotypes, which together ensure effective and sustainable management of this important disease of eggplants.

**Supplementary Information:**

The online version contains supplementary material available at 10.1186/s12870-024-05257-4.

## Background

*Solanum melongena L.*, commonly called eggplant or brinjal, holds a pivotal position in solanaceous vegetable crops, cultivated across the globe, primarily in subtropical and tropical regions. China is the top producer, contributing more than 60% of the world’s brinjal production, followed by India with 22.58%, while Egypt, Turkey and Indonesia are the other significant producers [3]. Being rich in vitamins, minerals, soluble and free-reducing sugars, proteins, phenols and anthocyanin and with its remarkable productivity and accessibility have earned it the endearing mark of “poor man’s vegetable” in Asian countries [43].

However, despite its prominence, eggplant cultivation is disturbed significantly due to biotic and abiotic stresses. Among them, little leaf disease, caused by a phytoplasma, emerged as a formidable rival, inflicting substantial economic losses [27, 41]. Infected plants exhibit distinctive characteristics such as the development of undersized leaves, excessive shoot proliferation, phyllody and stunted growth [37]. In India, the disease was first documented by Thomas and Krishnaswami in 1939, followed by a series of comprehensive investigations specifically on the biological aspects of disease development [18, 19, 27].

The etiology of eggplant little leaf disease in India has been firmly established through symptomatology, electron microscopy and PCR assays [4, 9, 19]. Furthermore, weed species *Datura stramonium*, *D. inoxia*, *Cannabis sativa* subsp. *sativa*, *Portulaca oleracea* and *P. grandiflora* were identified as alternate hosts for the phytoplasma [19, 39, 44] while leafhopper *Hishimonas phycitis* was predicted as the likely vectors for the disease transmission [4, 17, 19].

The current approach to managing little leaf disease relies heavily on controlling its insect vector population through pesticide applications and cultural practices [25]. However, these methods have significant environmental and economic drawbacks when host resistance emerges as a promising alternative [8]. However, the process of identifying resistant genotypes across diverse environments remains challenging and requires appropriate assessment techniques and analytical tools. Utilizing disease incidence percentages [16, 19] and the apparent rate of disease progress between two observations has offered solutions for determining resistance in genotypes. Moreover, the advent of additive main effect and multiplicative interaction (AMMI) analysis has provided an effective approach for studying the performance of genotypes in diverse environmental conditions, thereby enhancing our understanding of genotype-environment interactions (G x E) [28, 35, 36, 40].

Hence, in addition to the molecular characterisation of the phytoplasma strains and insect vectors responsible for disease transmission, in this study, we screened eggplant germplasm for resistance to little leaf disease and assessed the stability of the identified resistant genotypes across multiple environments through AMMI analysis.

## Results

### Symptomatology and disease incidence survey

**Fig. 1:**
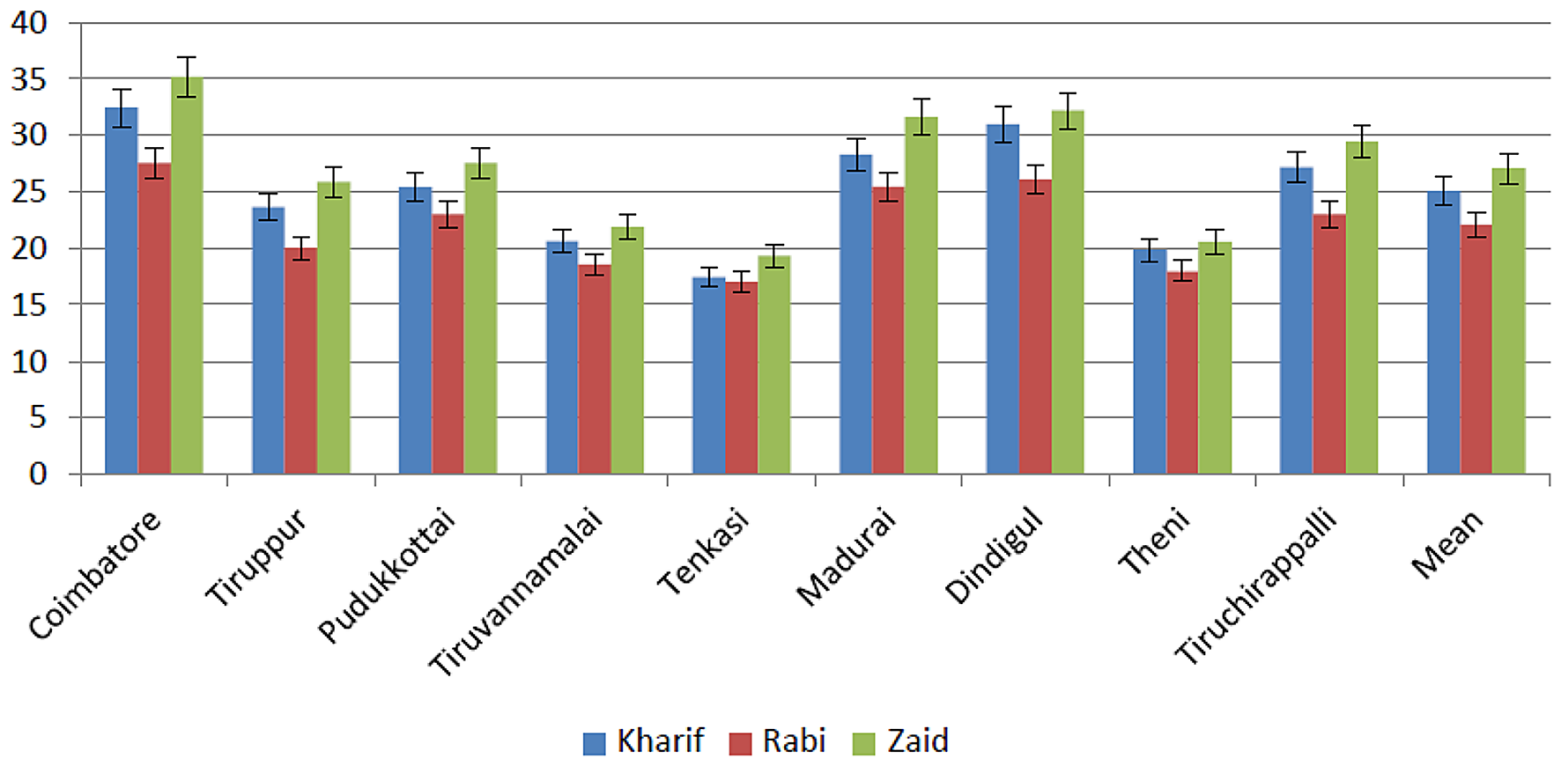
Incidence of brinjal little leaf in nine districts of Tamil Nadu state in India. Each bar is mean of three replications

**Fig. 2:**
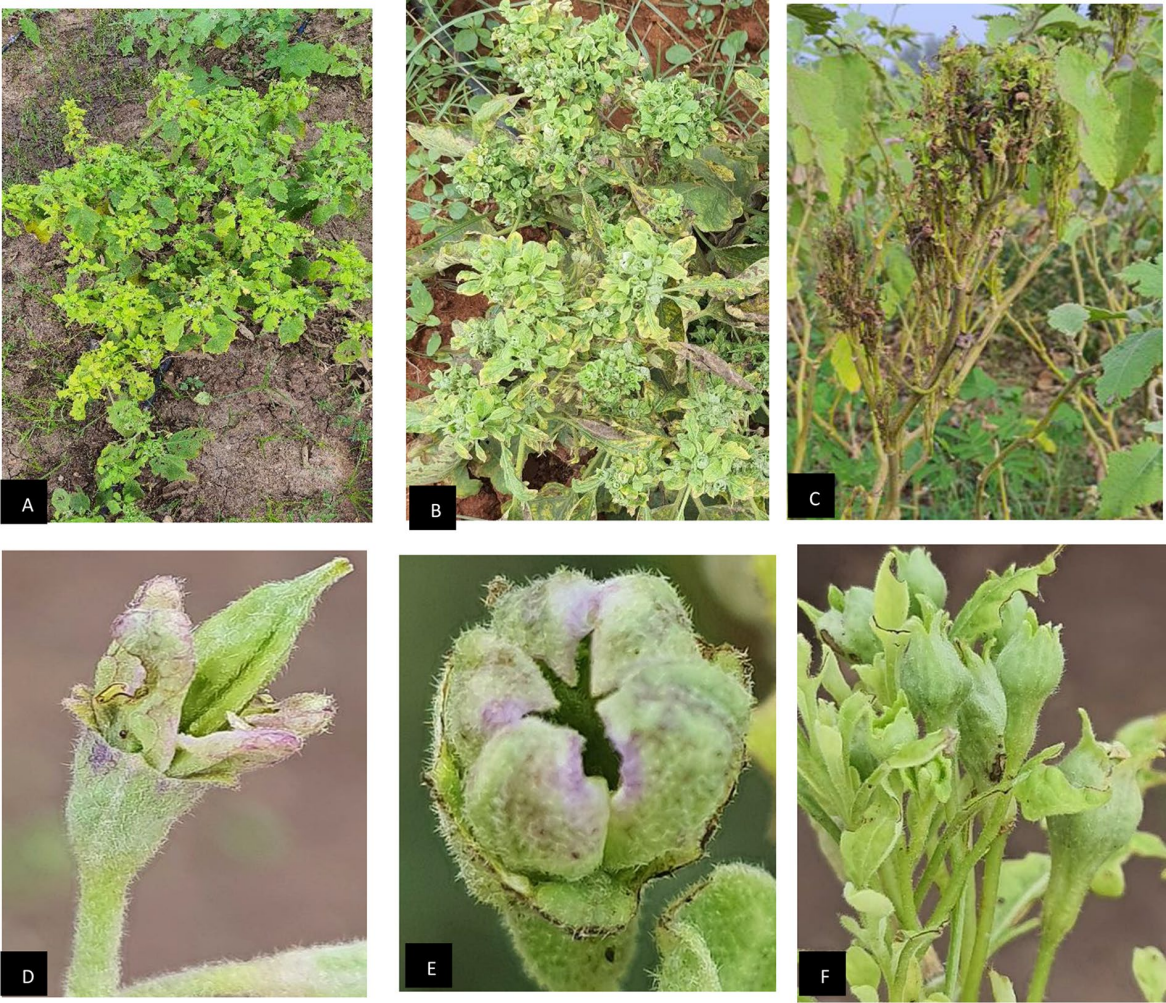
Typical phytoplasma symptoms were observed in infected eggplants. (**A**) little leaf, (**B**) chlorosis, (**C**) severe stunting and necrosis, (**D**) phyllody (**E**) virescence and (**F**) giant calyx

A comprehensive survey of eggplant fields across nine districts in Tamil Nadu state of India during 2021–2022 revealed varying incidences of phytoplasma infection. Based on the Percent Disease Incidence (PDI) mean value, the highest incidence (27.05%) occurred during the Zaid (March to June), followed by Kharif (June to November) (25.08%) and Rabi (November to March) (22.07%) seasons (Fig. 1). Phytoplasma-associated symptoms primarily included a drastic reduction in leaf size and excessive growth of axillary shoots, resulting in a bushy appearance of affected plants. Common symptoms observed included little leaf, virescence, phyllody, severe growth stunting, leaf chlorosis, and witches’ broom (Fig. 2A-F).

### Confirmation of phytoplasma infection through PCR amplification

Amplification of the nine individual symptomatic samples collected from nine districts of Tamil Nadu, India using phytoplasma-specific primer pairs (P1/P6) [10] amplified a 1.5 kb product (Supplementary figure S1). Subsequent evaluation using R16F2n/R16R2 [45] in the nested PCR assays yielded specific amplicons of 1.2 kb from each of the nine symptomatic eggplant plant samples (Supplementary figure S2). These results indicated the presence of phytoplasma in all evaluated symptomatic eggplant samples and sequenced for further confirmation. The sequence data was also submitted to NCBI Bank (Table 1). No amplification was observed in DNAs extracted from healthy plant samples used as negative controls in both the first round and nested PCR experiments with the same set of primers.

**Table 1:** Sample ID, GenBank accession numbers, Percentage ID with reference strain from data set as obtained by iPhyClassifier and ‘Candidatus Phytoplasma’ species assignment

Sr.No.	Sample ID	GenBank accession numbers	Percentage ID% with reference strain from data set	Phytoplasma classification based on iPhyClassifier
1	BLLP-1	ON870804	98.1% with AY390261	‘Candidatus Phytoplasma trifolii’-related strain.
2	BLLP-2	ON870839	97.8% with AY390261	‘Candidatus Phytoplasma trifolii’-related strain.
3	BLLP-3	ON870840	98.4% with AY390261	‘Candidatus Phytoplasma trifolii’-related strain.
4	BLLP-4	ON870841	98.1% with AY390261	‘Candidatus Phytoplasma trifolii’-related strain
5	BLLP-5	ON870842	98.2% with AY390261	‘Candidatus Phytoplasma trifolii’-related strain
6	BLLP-6	ON870843	98.2% with AY390261	‘Candidatus Phytoplasma trifolii’-related strain
7	BLLP-7	ON872227	97.7% with AY390261	‘Candidatus Phytoplasma trifolii’-related strain
8	BLLP-8	ON870844	97.8% with AY390261	Candidatus Phytoplasma trifolii’-related strain
9	BLLP-9	ON870845	97.4% with AY390261	Candidatus Phytoplasma trifolii’-related strain
10	Hp-CBE	ON870850	98.2% with AY390261	Candidatus Phytoplasma trifolii’-related strain

### Phytoplasma detection in insects by PCR assays

In the PCR analysis of the various insect species (10 insects per species) associated with eggplant, including *Amrasca biguttula biguttula*, *Hishimonus phycitis*, *Leucinodes arbonalis*, *Bemisia tabaci* and *Henosepilachna vigintioctopunctata*, 1.25 kb PCR amplicon was observed only from *H. phycitis*, confirming its prominent role as the vector for the disease transmission (Supplementary figure S3). The 16 S rRNA of *H. phycitis* was sequenced for further confirmation, and the sequence data was also submitted to GenBank (HP: Hp CBE; Accession No: ON870850). Additionally, greenhouse transmission assays were carried out utilizing positive *H. phycitis* populations. Following 2 months of feeding under the cage, 50% of brinjal plants in all replications exhibited characteristic little leaf disease symptoms, while control cages without leafhopper inoculation showed no signs of disease.

### BLAST and phylogenetic analyses of *H. phycitis* 16 S rDNA sequences

**Fig. 3:**
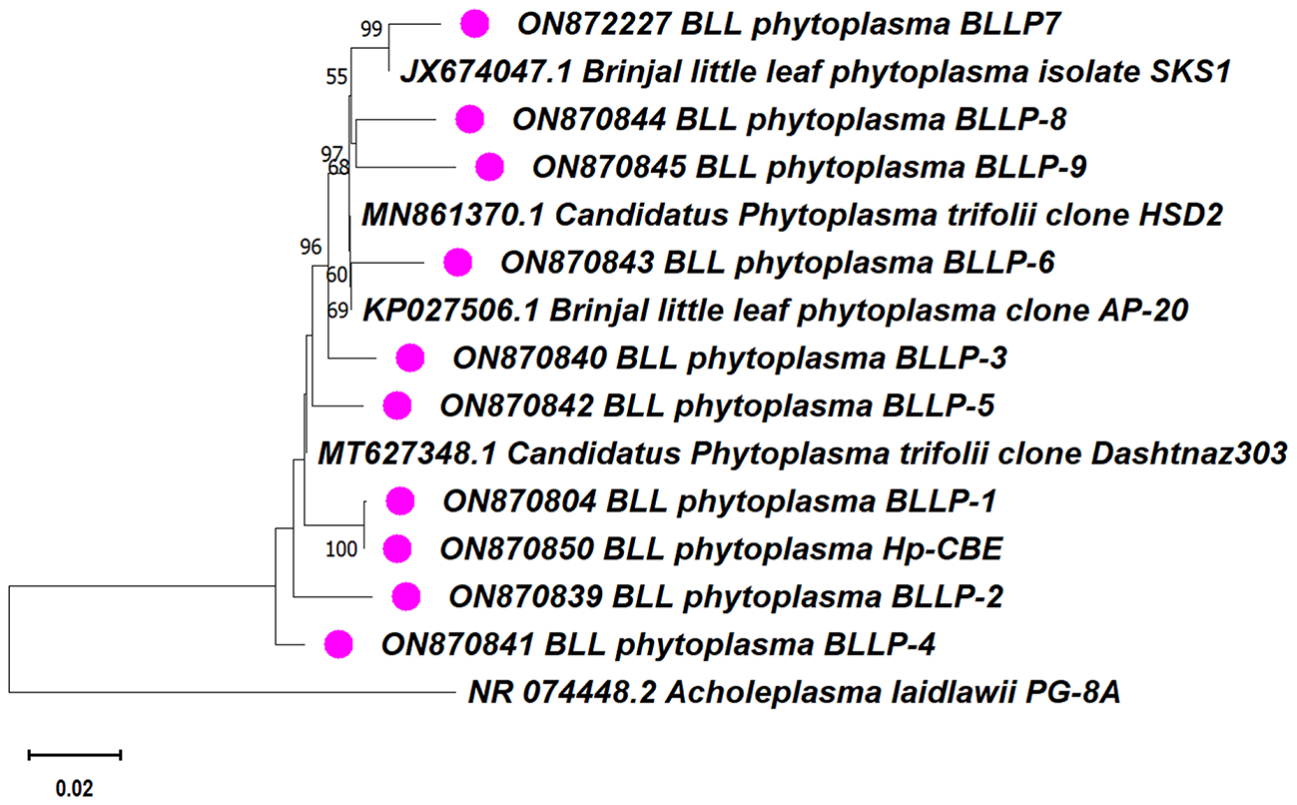
Phylogenetic tree based on 16SrDNA constructed by neighbour-joining method showing the relationships among eggplant, leafhopper and the reference phytoplasma strains

BLAST analysis of the 16S rRNA partial sequences obtained from all *H. phycitis* samples indicated similarity with the 16Sr VI group of Candidatus Phytoplasma trifolii. Subsequently, in the phylogenetic tree analysis, the 16S rRNA sequence from the eggplant little leaf isolates BLLP-1 to BLLP-9 and the *H. phycitis* isolate Hp - CBE clustered together with the reference strain Candidatus Phytoplasma trifolii, derived from clover (Fig. 3).

### Characteristics of *H. phycitis*.

**Fig. 4:**
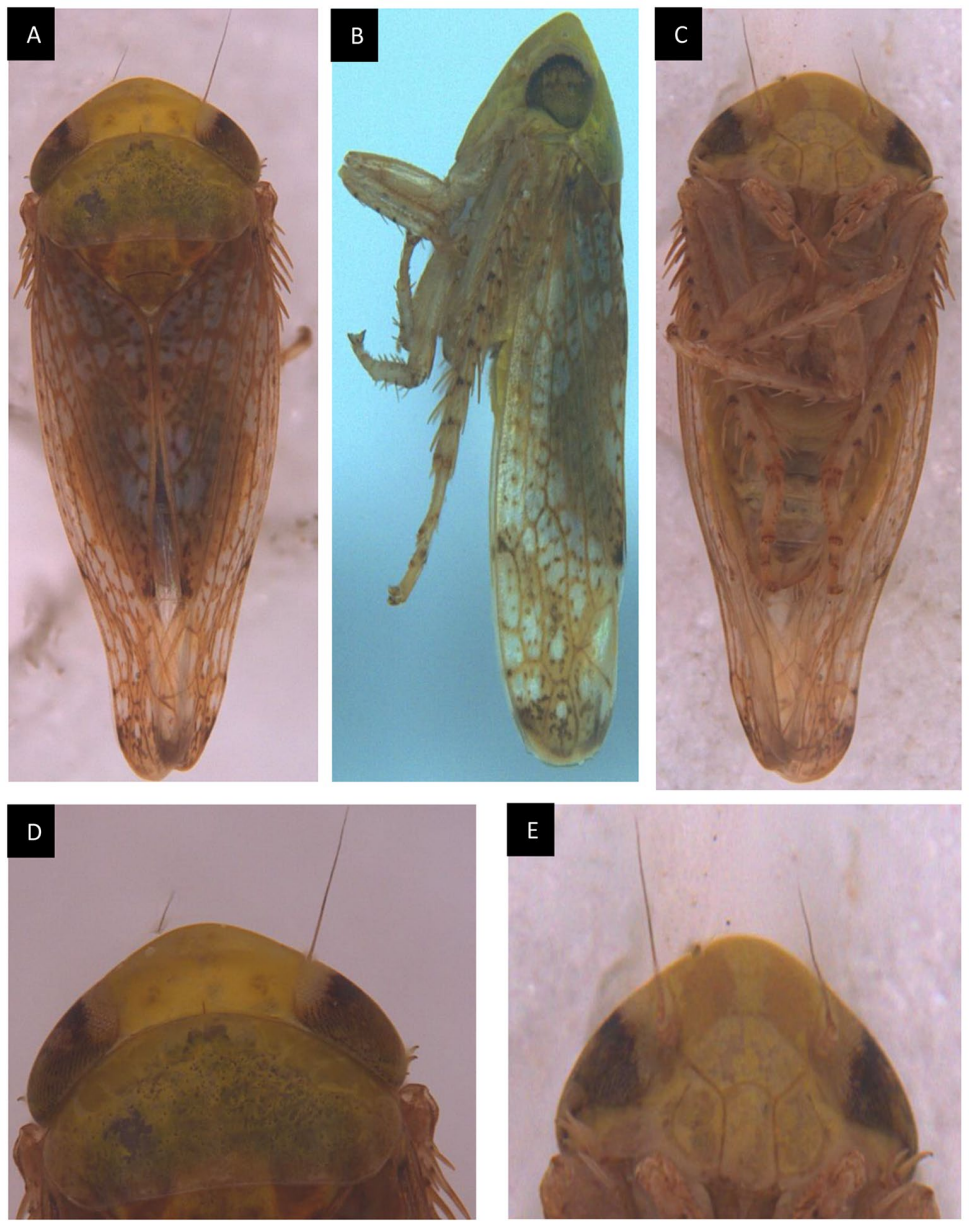
External Morphology of *Hishimonus phycitis* adult male: (**A**). Dorsal view, (**B**). Lateral view, (**C**). Ventral view, (**D**). Head with pronotum, dorsal view, (**E**). Head, Ventral view

*Hishimonus phycitis* was characterised at the species level in the Department of Agricultural Entomology, Tamil Nadu Agricultural University (TNAU), Coimbatore, India. Species-specific features consist of compound eyes black with a whitish apical margin and ocelli at the anterior margin of the vertex contiguous with compound eyes. The vertex is devoid of spots and the cypleal suture is distinct. The scutellum is triangular with black traverse suture in the middle with yellowish brown. Wings dark brown mottling spots are present all over the wing, and a black semi-circular spot is present at the commissural margin of each wing. when wings are folded at rest, this spot looks like a prominent circular spot. Pictographic representations of *H. phycitis* are given in (Fig. 4).

### Population dynamics of *H. phycitis*

Population dynamics of *H. phycitis* were investigated during the Kharif, Rabi, and Zaid seasons (Table 2). The population of *H. phycitis* reached a maximum during Zaid, followed by the Kharif and Rabi seasons. During the Kharif season, the population of *H. phycitis* showed positive correlations with maximum and minimum temperatures, morning and evening relative humidity, and wind speed, and negative correlations with rainfall. During the Rabi season, positive correlations with maximum temperature (28-30.5 °C) and wind speed (3.2-8 km/hr), while negative correlations with minimum temperature (19.5–27.5 °C), morning and evening relative humidity (84–91 & 45–79%) and rainfall (0.5–63 mm) were obvious. However, in the Zaid season, the population of *H. phycitis* displayed positive correlations with minimum temperature, evening relative humidity, rainfall, and wind speed, and negative correlations with maximum temperature and morning relative humidity (Table 3). The population dynamics of *H. phycitis* were also found to be influenced by weather parameters, accounting for 99, 95 and 96% of the variation (Table 4).

**Table 2:** Population dynamics of *Hishimonus phycitis* at Coimbatore during 2021–2022

Growth stages (DAT^#^)	Kharif		Rabi		Zaid	
30	0*		0		0	
40	0		0		0	
50	0		0		0	
60	0		0		2	
70	2		1		4	
80	4		3		5	
90	5		4		6	
100	6		5		8	
110	8		7		10	

^#^DAT - days after transplanting

*Number of *H. phycitis* adults per 10 sweeps

**Table 3:** Correlation coefficient (r) and regression equation *Hishimonus phycitis* vs. weather parameters for Kharif, Rabi and Zaid seasons

Weather parameters	Kharif	Rabi	Zaid
Maximum Temperature	0.42 y = -19.75 + 0.70 T. Max	0.38 y = -23.01 + 0.86 T. Max	-0.57 y = 40–1.08 T. Max
Minimum Temperature	0.24 y = -14.69 + 0.74 T. Min	-0.20 y = 11.48 − 0.42 T. Min	0.58 y = -12.01 + 0.69 T. Min
Morning Relative Humidity	0.33 y = -11.08 + 0.16 M. RH	-0.45 y = 53.56–0.59 M. RH	-0.23 y = 12.92–0.11 M. RH
Evening Relative Humidity	0.1 y = 1.09 + 0.02 E. RH	-0.80 y = 12.44–0.17 E.RH	0.58 y = -1.83 + 0.12 E.RH
Rainfall	-0.09 y = 2.87–0.875 RF	-0.48 y = 2.86–0.05 RF	0.62 y = 2.92 + 1.5 RF
Wind speed	0.32 y = 1.30 + 0.24 WS	0.23 y = 1.51 + 0.19 WS	0.74 y = -2.04 + 0.96 WS

T. Max-temperature maximum, T. Min-temperature minimum, M. RH-morning relative humidity, E. RH-evening relative humidity, RF-rainfall, WS-wind speed

**Table 4:** Multiple regression equation *Hishimonus phycitis* vs. total weather parameters for Kharif, Rabi and Zaid seasons

Season	Multiple regression equation	*R* square value
Kharif	y = -62.96 + 5.87 T. Max − 4.49 T. Min − 0.69 M. RH + 0.73 E. RH − 7.34 RF -0.007 WS	0.99
Rabi	y = -87.09 + 0.99 T. Max + 0.85 T. Min + 0.76 M. RH – 0.39 E. RH + 0.09 RF – 0.11 WS	0.95
Zaid	y = 7.87–0.25 Max + 1 T. Min – 0.25 M. RH + 0.009 E. RH + 0.51 RF + 0.21 WS	0.96

T. Max-temperature maximum, T. Min-temperature minimum, M. RH-morning relative humidity, E. RH-evening relative humidity, RF-rainfall, WS-wind speed

### Resistance responses of eggplant germplasm against little leaf disease

The experiment was conducted during the year 2021–2022 and aimed to identify resistance sources for little leaf disease in eggplant. None of the tested accessions were found to be free from little leaf disease incidence. Observations were statistically analysed and mean values were tabulated (Table 5). Among the 24 genotypes tested, CBE-SM-105 and CBE-SM-106 exhibited the lowest little leaf disease incidence (8.06%), followed by PLR 1 (9.10%), and CBE-SM-119 (18.24%), which had the highest incidence under natural conditions. In artificially inoculated conditions, a few entries, such as CBE-SM-105, CBE-SM-086, CBE-SM-104, CBE-SM-106, CBE-SM-108, and PLR-1, displayed moderately resistant reactions, while the remaining entries were susceptible and highly susceptible (Table 6).

**Table 5:** Reaction of eggplant genotypes to little leaf disease under artificial and field conditions

			Little leaf disease incidence (%)								
Genotype code	Genotype	Artificial screening (%)	Natural condition						Mean	PCA 1	PCA 2
			Kharif		Rabi		Zaid				
			**R1**	**R2**	**R1**	**R2**	**R1**	**R2**			
G1	C0-2	46.67	12.11	12.54	12.18	12.59	12.48	12.84	12.53	-0.01	-0.25
G2	CBE-SM-112	33.33	10.21	10.23	9.87	10.19	10.06	10.42	10.15	0.13	-0.02
G3	CBE-SM-113	53.33	13.58	13.59	13.23	13.61	13.70	14.06	13.64	-0.05	-0.06
G4	CBE-SM-114	60.00	14.68	14.79	14.43	13.95	14.89	15.25	14.66	-0.18	0.26
G5	CBE-SM-115	60.00	15.56	15.65	15.29	15.53	15.54	15.90	15.58	0.08	-0.05
G6	CBE-SM-116	66.67	16.58	15.75	15.39	15.63	15.51	15.87	15.63	0.32	0.30
G7	CBE-SM-117	40.00	10.39	10.94	10.58	10.23	10.31	10.67	10.55	0.25	0.04
G8	CBE-SM-118	66.67	16.19	16.30	15.94	16.42	15.38	15.74	15.95	0.63	-0.18
G9	CBE-SM-119	73.33	18.04	18.26	17.90	18.21	18.24	18.60	18.24	0.02	-0.12
G10	CBE-SM-006	40.00	10.05	10.17	9.81	10.11	10.60	10.96	10.33	-0.36	-0.03
G11	CBE-SM-083	53.33	12.77	12.85	12.49	12.22	12.92	13.28	12.75	-0.15	0.21
G12	CBE-SM-084	46.67	11.96	11.95	11.59	11.95	12.02	12.38	11.98	-0.03	-0.04
G13	CBE-SM-085	46.67	11.76	11.43	11.07	11.50	11.47	11.83	11.46	0.07	0.08
G14	CBE-SM-086	20.00	9.42	9.42	9.06	9.42	9.42	9.78	9.42	0.00	-0.02
G15	CBE-SM-093	26.67	10.08	9.69	9.33	9.99	10.04	10.40	9.89	-0.13	0.02
G16	CBE-SM-104	20.00	9.11	9.18	8.82	8.89	9.14	9.50	9.11	-0.05	0.10
G17	CBE-SM-105	13.33	8.06	8.15	7.79	8.13	8.14	8.50	8.06	-0.03	-0.04
G18	CBE-SM-106	20.00	8.06	7.51	7.15	7.57	7.63	7.99	8.06	0.01	0.26
G19	CBE-SM-107	40.00	11.27	11.06	10.70	11.30	11.33	11.69	11.27	-0.10	-0.04
G20	CBE-SM-108	20.00	9.33	9.26	8.90	9.30	9.03	9.39	9.33	0.21	-0.01
G21	CBE-SM-109	33.33	10.04	10.57	10.21	10.64	10.72	11.08	10.04	-0.20	-0.30
G22	CBE-SM-110	53.33	12.72	12.71	12.35	12.73	12.79	13.15	12.72	-0.02	-0.04
G23	CBE-SM-111	26.67	9.51	9.48	9.12	9.54	9.74	10.10	9.51	-0.19	-0.02
G24	PLR-1	20.00	9.10	9.41	8.85	9.37	9.45	10.01	9.10	-0.23	-0.04

**Table 6:** Resistance categories of different eggplant genotypes against little leaf disease

Genotypes		Disease incidence	Category
Natural condition	Artificially inoculated condition		
CBE-SM-105, CBE-SM-106, PLR-1, CBE-SM-104, CBE-SM-108, CBE-SM-086, CBE-SM-111, CBE-SM-093		0.1–10.0	Resistant
CBE-SM-109, CBE-SM-112, CBE-SM-006, CBE-SM-117, CBE-SM-107, CBE-SM-085, CBE-SM-084, C0-2, CBE-SM-110, CBE-SM-083, CBE-SM-113, CBE-SM-114, CBE-SM-115, CBE-SM-116, CBE-SM-118, CBE-SM-119	CBE-SM-105, CBE-SM-086, CBE-SM-104, CBE-SM-106, CBE-SM-108, PLR-1	10.1–20.0	Moderately resistant
	CBE-SM-093, CBE-SM-111, CBE-SM-112, CBE-SM-109, CBE-SM-117, CBE-SM-006, CBE-SM-107, C0-2, CBE-SM-084, CBE-SM-085	20.1–50.0	Susceptible
	CBE-SM-113, CBE-SM-083, CBE-SM-110, CBE-SM-114, CBE-SM-115, CBE-SM-116, CBE-SM-118, CBE-SM-119	> 50.0	Highly susceptible

### AMMI analysis

**Fig. 5:**
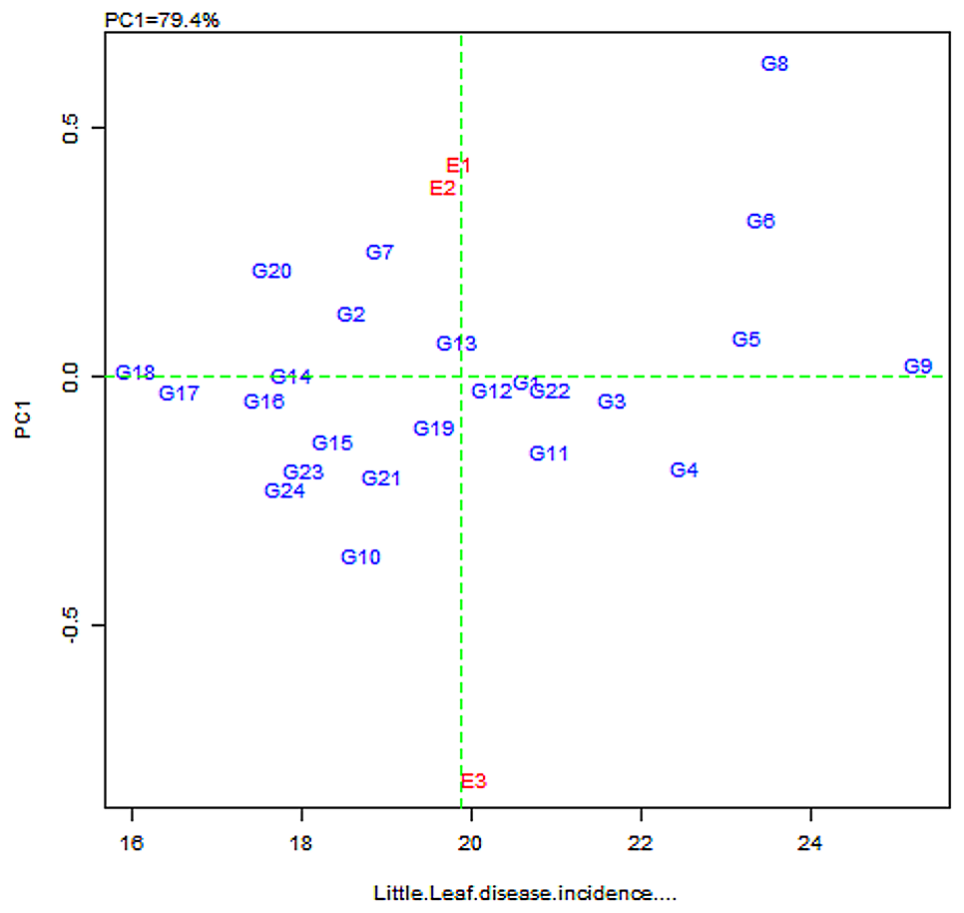
AMMI1 Biplot display for mean little leaf disease incidence (%) and IPCA 1 scores of the 24 genotypes (G) tested across three environments (E). E1: Kharif, E2: Rabi, E3: Zaid

Genotypes such as G18 (CBE-SM-106), G17 (CBE-SM-105), G16 (CBE-SM-104), G14 (CBE-SM-086), G12 (CBE-SM-084), G1(C0-2), G22 (CBE -SM-110), G3 (CBE-SM-113) and G9 (CBE-SM-119), were plotted away from the x axis in AMMI I biplot which shows low interaction or consistent performance across the environment. However, the lines G18 (CBE-SM-106) and G17 (CBE-SM-105) are highly resistant, since they had low mean values for disease incidence. G9 (CBE-SM-119) and G5 (CBE-SM-115) lines with high mean values were considered as susceptible to little leaf. On the other hand, the genotypes on a horizontal line have similar interaction patterns, such as G12 (CBE-SM-084) and G22 (CBE-SM-110) (Fig. 5).

Genotypes or environments on the right side of the y-axis have higher mean disease incidence, while those placed on the left side have a lower mean. As a result, it is quite evident from the AMMI biplot that, the genotypes G9 (CBE-SM-119), G5 (CBE-SM-115), G4 (CBE-SM-114), G3 (CBE-SM-113), G11 (CBE-SM-083), G22 (CBE-SM-110), G1 (C0-2) and G12 (CBE-SM-084) had a low level of resistance. In contrast, G18 (CBE-SM-106), G17 (CBE-SM-105), G16 (CBE-SM-104), G14 (CBE-SM-086) and G20 (CBE-SM-108) were highly resistant to the little leaf of eggplant. However, in the presence of environmental influence, E3 on the right-hand side are favourable for disease incidence, while E1 and E2 on the opposite side were slightly less favourable.

**Fig. 6:**
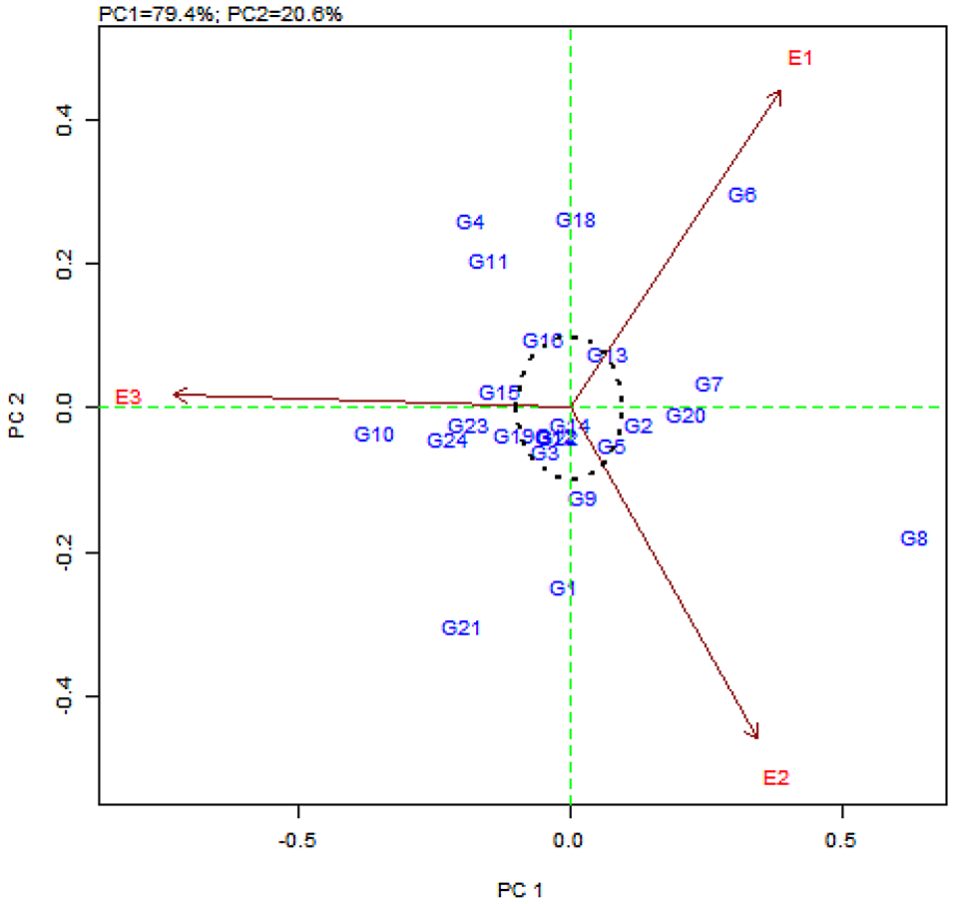
AMMI2 biplot for 24 genotypes (G) and three environments (E) displayed on the first and second principal component axis (IPAC 1 vs. IPAC 2). E1: Kharif, E2: Rabi, E3: Zaid

The AMMI2 biplot derived using PC1 and PC2 shows interaction effect, and the genotypes plotted near the origin are considered to be stable. Thus G14 (CBE-SM-086), G12 (CBE-SM-084), G22 (CBE-SM-110), G3 (CBE-SM-113), G13 (CBE-SM-085), G5 (CBE-SM-115), G19 (CBE-SM-107) and G16 (CBE-SM-104) are stable for resistant to little leaf disease. The lines that are far away from the origin, such as G8 (CBE-SM-118), G6 (CBE-SM-116) and G21 (CBE-SM-109), are less stable for disease incidence. The environments E1, E2 and E3 all had higher level projections from the origin, indicating that all environments are highly discriminative for disease incidence (Fig. 6). Also, E2 observed with low PCA1 scores had low interactions and great stability. However, the high PCA1 scores in E3 and E1 showed high interaction, stability and adaptation to specific environments.

A significantly larger proportion of genotypes recorded low IPCA1 scores and showed a small interaction effect, which led to the clustering of the genotypes on the biplot. Genotypes G8 (CBE-SM-118) recorded the highest IPCA1 score of 0.6 followed by genotypes viz. G6 (CBE-SM-116), G7 (CBE-SM-117), G20 (CBE-SM-108) and G2 (CBE-SM-112). This indicated that the high interaction effect of genotypes had specific stability and adoption to environments (Tables 7 and 8).

**Table 7:** Environments

Sl. No	Environment	Environment description	PCA 1	PCA 2
1	E1	Kharif	-0.58361	0.433797
2	E2	Rabi	-0.58492	0.3618
3	E3	Zaid	-0.56326	-0.82518

**Table 8:** AMMI analysis of variance (ANOVA) for G x E interactions

Source	df	SS	MS	%
PC1	24	0.966422	0.040268	79.4
PC2	22	0.250433	0.011383	20.6
PC3	20	0.000000	0.000000	0.0

### GGE biplot

**Fig. 7:**
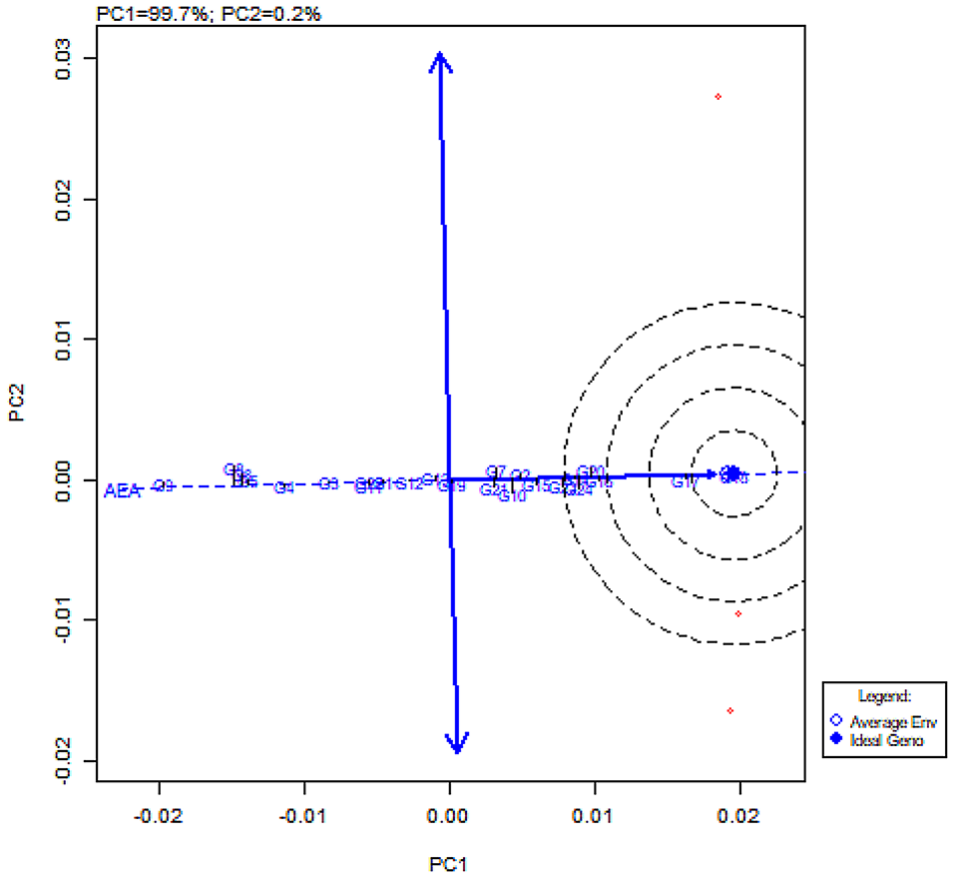
Genotype view of GGE biplot analysis of genotypes relative to ideal genotype (centre of the concentric circles)

**Fig. 8:**
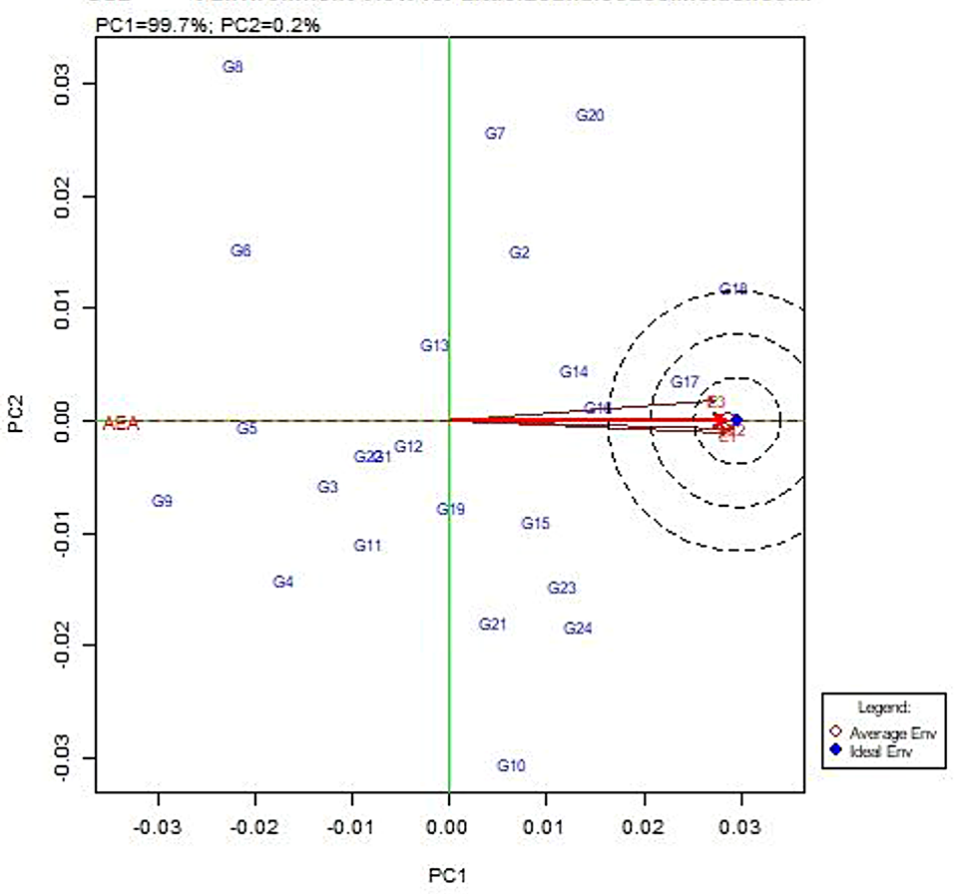
Environment view of GGE biplot analysis: ranking of environments relative to an ideal test location (represented by the centre of the concentric circles); E1: Kharif, E2: Rabi, E3: Zaid

**Fig. 9:**
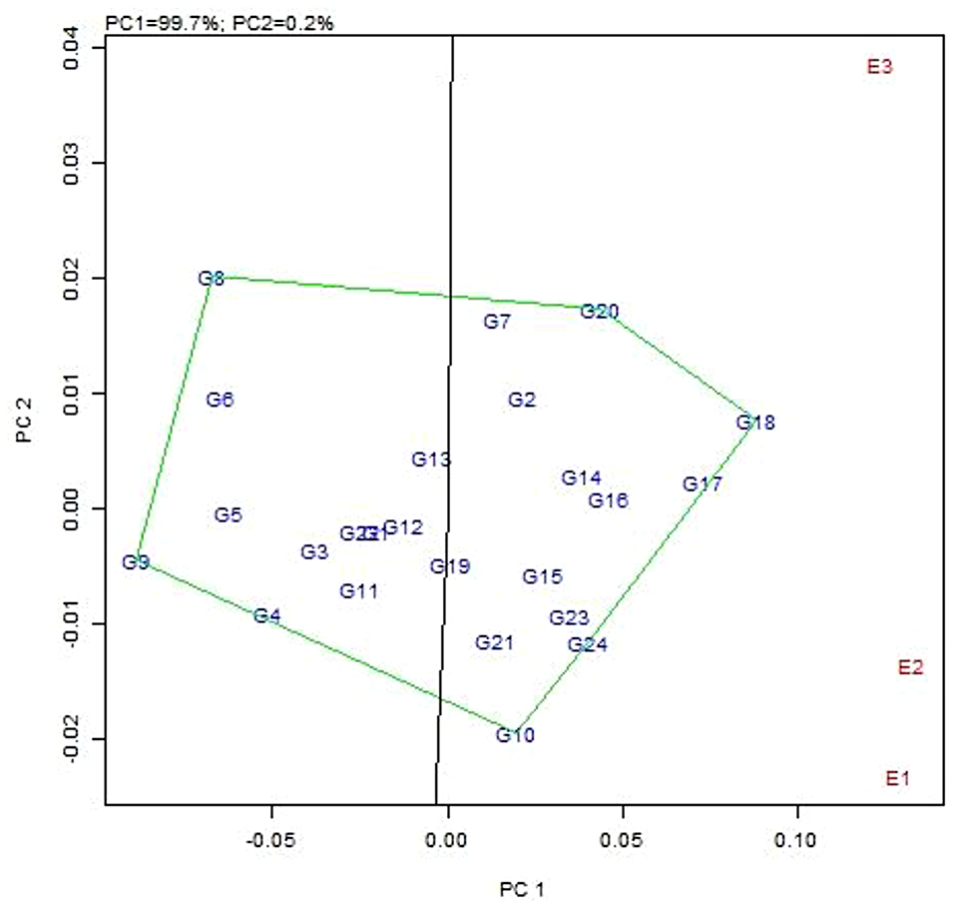
The which-won-where view of the GGE biplot showing which genotypes performed best in which environments: E1: Kharif, E2: Rabi, E3: Zaid

GGE biplot analysis was used to study the performance of genotypes in each environment. The centre of the concentric circles defines the ideal genotype, while those closer to this point are regarded as near the ideal genotype. In this study, the clusters of genotype present in the centre of the concentric ring show the ideal genotype, whereas G17 (CBE-SM-105), G16 (CBE-SM-104) and G24 (PLR-1) are near the ideal genotype (Fig. 7). In the average environment coordination (AEC) analysis, E3 (Zaid) appeared to be the most discriminating environment when compared with E1 (Kharif) and E2 (Rabi) (Fig. 8). The analysis also showed that all environments were ideal for little leaf disease screening. Genotypes clustered closely to the biplot origin of G17 (CBE-SM-105) were similar in little leaf disease-resistant status and stability. The genotypes scattered from the origin were considered to vary in little leaf disease-resistant status and were found to be less stable. The which-won-where biplot was employed to identify the best genotypes suited to various test environments, where G18 (CBE-SM-106) recorded the best-performing genotype in all test environments (Fig. 9).

## Discussion

Eggplant or brinjal is a globally important vegetable crop, cultivated year-round in tropical regions. Among the numerous challenges faced by eggplant cultivation, the emergence of eggplant little leaf disease, caused by phytoplasma, poses a significant threat, potentially leading to 100% crop loss [19]. Phytoplasmas are non-helical bacteria devoid of cell walls, known to affect thousands of plant species [15, 21, 23, 26, 38, 42]. They are considered one of the most molecularly enigmatic genera of plant pathogens due to their inability to be cultured in vitro, irregular host distribution, and the challenges associated with serology and electron microscopy-based identification. However, biotechnological interventions, such as nested PCR targeting the 16S rRNA gene, have overcome these limitations, aiding in the identification and classification of phytoplasmas [7, 29]. Phytoplasmas are increasingly recognized as emerging plant pathogens with significant economic impacts, comparable to viruses.

Eggplant little leaf disease was characterized by a conspicuous reduction in leaf size, leading to the gradual shrinkage of newly produced leaves. This was accompanied by a notable reduction in petiole size, resulting in slender, hairless, yellow, and closely appressed leaves with shorter internodes. Axillary buds exhibited excessive growth, developing into stunted branches with tiny leaves, giving the plants a rosette-like appearance. Phyllody, transforming floral structures into vegetative-like structures, was another typical symptom. In severe cases, plants became sterile as flowers remained green, upright, and generally unproductive [24].

These symptoms closely resembled those reported in previous studies of phytoplasma-related diseases. For instance, reduced leaves, virescence, and witches’ broom symptoms in phytoplasma-infected Catharanthus plants in Egypt [32]. Symptoms include stunting, yellow to purple leaves, severe lateral bud proliferation, hypertrophic calyx development, virescence, suppression of ovary and anther production, and petal fusion [33]. Increased branching, reduction in leaf size, and phyllody were also noticed in infected plants. Fruiting was also affected, with malformed fruits and shrivelled seeds in late-stage infections [27].

Molecular detection of phytoplasma in this study utilized nested PCR assays targeting the 16S rRNA gene. The 16S rRNA gene region was amplified with universal primers P1/P6, followed by nested primer pair R16F2n and R16R2, resulting in DNA amplification of approximately 1.5 and 1.2 kb, respectively. These findings align with previous research, including the work of [19], who reported similar results in both plants and vectors, using the same primer pairs. The highly conserved 16S rRNA gene region was employed as the primary molecular tool for phytoplasma identification, genotyping, taxonomic assignment, and group/subgroup classification [22]. All nine phytoplasma samples from plants and one from vectors in this study were identified as ‘Candidatus Phytoplasma trifolii’ related strains, focusing on the conserved region of the 16S rDNA. This study validates the findings of [19], who associated ‘Candidatus Phytoplasma trifolii’ with eggplant little leaf. Also, the phytoplasma associated with eggplant little leaf disease belongs to the 16SrVI clover proliferation phytoplasma group [4].

Phytoplasmas primarily reside in the sieve cells of phloem tissue, inducing various disease symptoms and hormonal imbalances. Insect vectors acquire phytoplasmas from infected plants while feeding on phloem sap. These vectors transport phytoplasmas through their gastrointestinal tracts, hemocoel, and various internal organs, ultimately transmitting them to the plant phloem via their salivary glands [11]. Two leafhopper species, *H. phycitis* and *A. biguttula biguttula*, have been implicated in the transmission of little leaf in eggplant. However, their efficiency as vectors may vary. In the eggplant ecosystem of West Bengal [25], reported the presence of both insect vectors, but only *H. phycitis* was confirmed as a carrier and natural vector of the 16SrVI-D subgroup of phytoplasmas using nested PCR assays, sequence comparison, phylogeny, virtual RFLP analysis, and transmission assays. This study reaffirms the role of *H. phycitis* as a vector for eggplant little leaf disease [25]. The phloem-sap feeding behaviour of *H. phycitis* likely contributes to the transmission of eggplant little leaf [19].

A total of 24 genotypes of germplasm were evaluated for the little leaf resistance in which CBE-SM-105 and CBE-SM-106 were moderately resistant also reported earlier [6, 31]. The resistant genotypes could be used as a parent for developing a little leaf-resistant variety to promote cultivation in disease-prone and endemic areas of Tamil Nadu, India. The AMMI analysis model first accounts for the main effect, while the subsequent PCA predicted the interactions [13]. Previous findings also confirmed that AMMI analysis is an efficient tool for screening the resistance level of various genotypes [28, 36]. The additional years of testing the germplasm could confirm a specific test location and environment that will be most representative, discriminating, and ideal. Further research at the molecular level might be useful in identifying the genes and proteins responsible for resistance and susceptibility genotypes. A genotype with higher mean performance and stability across the test locations is said to be an ideal genotype. The which-won-where biplot recorded G18 (CBE-SM-106) as ideal genotypes based on the presence in the centre of the concentric ring. Similarly, a study on genotype G4 (Karuna) was closer to the centre of the concentric ring, followed by G1 (Harasoya) and G5 (Himso) in which Karuna may be chosen as an ideal genotype [30]. Cumulatively, PCA1 and PCA2 contributed to 60% of the total interaction. Thus, the interaction between the 24 genotypes across three test environments, in which the best genotype was predicted by the first two principal compounds. Sufficient research reports have also stated that the genotype interactions were precisely presented by two PCAs [28, 35].

Eggplant cultivation in India often coincides with the cultivation of various other crops, raising concerns about the natural spread of phytoplasmas across different plant species. Efficient vector species facilitate the transmission of phytoplasmas between eggplants and other plants. Thus, it is crucial to comprehensively assess the epidemiological factors contributing to the natural dissemination of phytoplasmas associated with eggplant little leaf disease in India [19]. This study substantiates the presence of a new natural vector and identifies resistant and susceptible genotypes harbouring phytoplasma. Eggplant little leaf disease poses a significant challenge in regions where eggplant is cultivated in India. To effectively combat this disease, a multifaceted approach is needed, including the cultivation of resistant eggplant cultivars, vector management, and regulation of natural hosts [37]. Identifying this vector and the associated conditions for phytoplasma occurrence, along with recognising potential genotypes, as reported in this study, opens new avenues for research. This information holds promise for developing more effective strategies to manage eggplant little leaf disease in India.

## Conclusion

In conclusion, this comprehensive study of eggplant little leaf disease, caused by phytoplasma, in Tamil Nadu, India, has provided valuable insights into the symptomatology, molecular detection, phytoplasma characterization, vector species, and evaluation of germplasm resistance. The disease’s symptoms, including leaf reduction, petiole thinning, axillary bud proliferation, and phyllody, closely resemble those observed in phytoplasma-related diseases in other plant species, aiding in its early identification. Molecular detection using nested PCR targeting the 16S rRNA gene confirmed the presence of ‘Candidatus Phytoplasma trifolii’ related strains, facilitating precise diagnosis and taxonomic assignment. The study reaffirmed the role of *H. phycitis* as a vector for eggplant little leaf disease and identified potential resistant genotypes, providing valuable resources for breeding programs. These findings have significant implications for managing eggplant little leaf disease in India and underscore the importance of a multifaceted approach, including developing resistant cultivars and vector control, to ensure the sustainability of eggplant cultivation in the region.

## Methods

### Disease survey and collection of samples

Between May to September 2022, leaf samples in triplicate were collected from symptomatic eggplant plants located in nine districts of Tamil Nadu state in India with the farmers’ permission. Samples were collected from 45 to 60 days old eggplant crops where a minimum of three to four samples were from diseased plants when two samples from healthy plants served as the negative control.

### DNA extraction and PCR assays

Genomic DNA from leaf samples, as well as for leafhopper species, whiteflies, hadda beetle, and shoot and fruit borer larvae, were extracted using the CTAB method outlined by [1]. Amplification of phytoplasma ribosomal DNA was achieved using the phytoplasma-universal primer (P1/P6) was designed on basis of 16S rDNA sequence which amplified a 1.5 kb product at initial denaturation 94ºC for 2 min, followed by 35 cycles of denaturation at 94ºC for 1 min, annealing at 55ºC for 1 min, extension at 72ºC for 1 min and final extension 72ºC for 10 min [10], Subsequent evaluation using R16F2n/R16R2 in the nested PCR assays by PCR products amplified by the universal primers P1/P6 were diluted 0 to 50 times in sterile deionized water and used as templates which yielded a specific amplicons of 1.2 kb at initial denaturation 94ºC for 2 min, followed by 35 cycles of denaturation at 94ºC for 1 min, annealing at 50ºC for 1 min, extension at 72ºC for 1 min and final extension 72ºC for 10 min [45]. For the nested PCR, the product from the first round of PCR assay was diluted to 1:4 ratio with sterile water and 2 µl of the diluted product was used as template. Subsequently, 5 µl of the nested PCR product were subjected to electrophoresis in a 1.0% (w/v) agarose gel, stained with ethidium bromide, and visualized under a UV transilluminator.

### Sequencing, pairwise sequence comparison, and phylogenetic analysis

PCR products of R16F2n/R16R2 were outsourced and sequenced bidirectionally at Eurofins Genomics India Pvt Ltd, Bangalore, India. The BioEdit sequence alignment editor tool was employed to assemble and generate consensus sequences by aligning sequences in both forward and reverse directions [14]. The 16S rRNA gene sequences of the samples were analyzed using the iPhy Classifier program (http://www.plantpathology.ba.ars.usda.gov/cgibin/resource/iphyclassifier.cgi), and the consensus sequences were submitted to GenBank. Additionally, 16S rRNA gene sequences of various phytoplasma groups were obtained from GenBank. Sequence homology percentages with other phytoplasma isolates were noted and subsequently utilized to construct a phylogenetic tree. Sequence alignment was performed using the CLUSTALW2 algorithm [20], and the phylogenetic tree was constructed using the neighbour-joining (NJ) method with MEGA version 11 [46], supported by 1000 bootstrap replications. The phylogenetic tree was rooted using the *Acholeplasma laidlawii* phytoplasma 16S rDNA sequence (Ac. No. NR074448).

### Insect sampling, identification, and population dynamics

The observation of *the H. phycitis* population involved counting the number of leafhoppers per 10 sweeps. The experiments spanned the Kharif, Rabi, and Zaid seasons of 2021–2022. Weather parameter data, including temperature, rainfall, relative humidity, daylight hours, and wind velocity, for the three seasons were obtained from the Agro Climate Research Centre, TNAU, Coimbatore, Tamil Nadu, India (Tables 9, 10 and 11). The collected insects were preserved in plastic vials containing 70% ethanol and stored at 4ºC for subsequent analysis. Taxonomic identification of the collected insects was carried out by the Department of Entomology, TNAU, Coimbatore, India. In accordance with Kumar et al.‘s protocol [18], transmission assays were conducted using the positive leafhopper samples. Five plants were maintained in both treated and control cages for the experiment.

**Table 9:** Meteorological data during the crop growth period (Kharif 2021–2022)

Day	SMW	Max. (Temp) °C	Min. (Temp) °C	Relative humidity (%) (Morning)	Relative humidity (%) (Evening)	Rainfall (mm)	Wind speed (km/hr)
Jul-02	27	34	24.5	84	45	0	9.6
12	29	29	23	70	62	0	0.4
22	30	30	23.5	78	64	0	1.5
Aug-01	31	33.5	22.2	91	42	0	10.3
11	33	32	23	86	66	1	3.7
21	34	31	24	87	74	0	3.4
31	36	32	22.5	85	56	0	5.4
Sep-10	37	33	22.5	85	50	0	11.1
20	38	34.5	25	85	56	0	8.2

**Table 10:** Meteorological data during the crop growth period (Rabi 2021–2022)

Day	SMW	Max. (Temp) °C	Min. (Temp) °C	Relative humidity (%) (Morning)	Relative humidity (%) (Evening)	Rainfall (mm)	Wind speed (km/hr)
Nov-08	46	28.5	21.5	86	79	63	3.2
18	47	30.5	21.5	86	79	57	3.3
28	48	27.5	23	91	72	0.5	5.8
Dec-08	50	29.5	22.5	86	65	0	4.8
18	51	29.5	19.5	85	57	0	4.5
28	53	28	20	85	45	0	6.8
Jan-07	2	28.5	22	84	49	0	4.6
17	3	29.7	22.6	86	55	0	8
27	5	31	20.5	85	47	0	3.3

**Table 11:** Meteorological data during the crop growth period (Zaid 2021–2022)

Day	SMW	Max. (Temp) °C	Min. (Temp) °C	Relative humidity % (Morning)	Relative humidity % (Evening)	Rainfall (mm)	Wind speed (km/hr)
Mar-03	10	33.5	17.5	73	21	0	6.6
13	11	35.2	18.2	70	24	0	4.8
23	13	34	23	86	44	0	3.2
Apr-02	15	35	24.5	88	45	0	4.7
12	16	33.5	24.5	86	78	1.2	3.5
22	17	35	26	81	46	0	6.1
May-02	19	34.5	26	84	44	0	4.9
12	20	29	23	70	50	0	10.8
22	21	32.5	24	72	60	4.5	10.8

### Germplasm evaluation for resistance to little leaf disease

Germplasm for resistance evaluation comprised 22 accessions and two check varieties, Co2 and PLR1. Of these, three were interspecific hybrid derivatives collected and maintained at the Department of Vegetable Crops, Horticultural College and Research Institute, TNAU, Coimbatore. The evaluation was conducted from February to June 2022 in a randomized block design and with three replications. Observations were recorded for 12 different traits from five randomly selected plants of each replication. The mean percentage of disease incidence was calculated based on the ratio of diseased to total plants across all replications. Five categories were established based on percent disease incidence: (i) immune (0%), (ii) resistant (0.1–10%), (iii) moderately resistant (10.1–20%), (iv) susceptible (20.1–50%), and (v) highly susceptible (> 50%) [8]. Germplasm was also screened for resistance to little leaf disease under artificial conditions using an insect-proof net house. Fifteen plants per line were raised in 25 cm pots, and each plant was inoculated with phytoplasma through side grafting using young shoots from diseased plants [8].

### AMMI and genotype by environment (G x E) analyses

AMMI statistical model and computational methods were used in this study as described in [5, 12, 28, 48]. The Analysis of variance (ANOVA) was generated using the computer software program MATMODEL version 3.0 [13]. The ‘genotype and genotype by environment’ (GGE) biplot analysis was utilized to check the stability of genotypes over the Kharif, Rabi and Zaid seasons [34, 47]. The AMMI biplot and PC analysis were carried out using Plant Breeding Tools Version 1.4 to find the susceptibility of the genotypes. As the data contains zero due to non-germination (NG) of seeds, 0.05 has been included for all the individual observations so that the results can be directly used with the biplot graph generated.

### Electronic supplementary material

Below is the link to the electronic supplementary material.

Supplementary Material 1

## Data Availability

The 16S rRNA of *H. phycitis* generated during the current study is available in NCBI GenBank (Accession No: ON870850).
