# Supplementary material for: Comprehensive analysis of little leaf disease incidence and resistance in eggplant
Source: BMC Plant Biol. 2024 Jun 18;24:576. doi: 10.1186/s12870-024-05257-4 (PMC11184749; doi:10.1186/s12870-024-05257-4)
Supplement: Supplementary file 1 — Supplementary Material 1 [file 12870_2024_5257_MOESM1_ESM.docx]

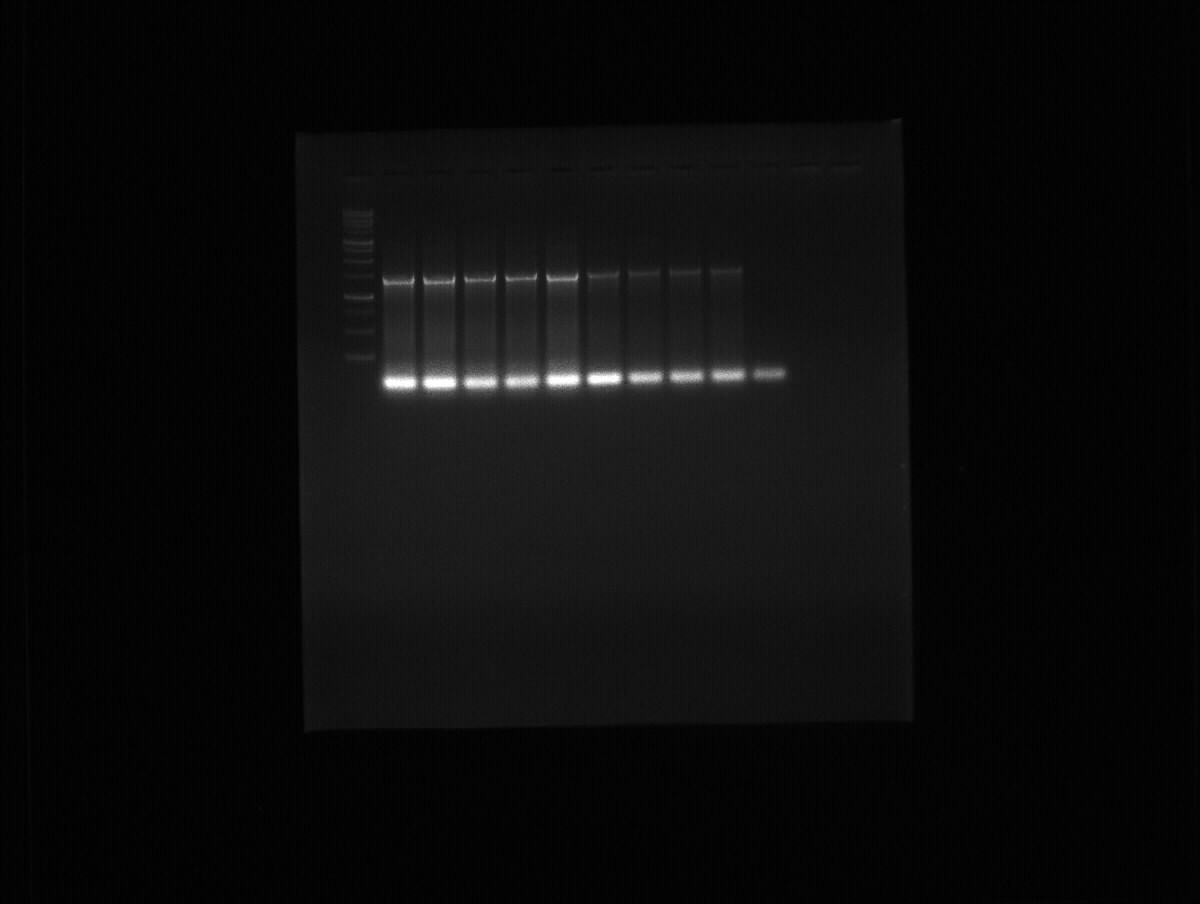


M L1 L2 L3 L4 L5 L6 L7 L8 L9 L10

~1.5 kb

**Supplementary figure S1.** Direct PCR assay results of phytoplasma DNA amplification from eggplant little leaf and phyllody plants with primer pair P1/P6; M: 1Kb ladder, Lane 1: BLLP-1, Lane 2: BLLP-2, Lane 3: BLLP-3, Lane 4: BLLP-4, Lane 5: BLLP-5, Lane 6: BLLP-6, Lane 7: BLLP-7, Lane 8: BLLP-8, Lane 9: BLLP-9 and Lane 10: Negative control


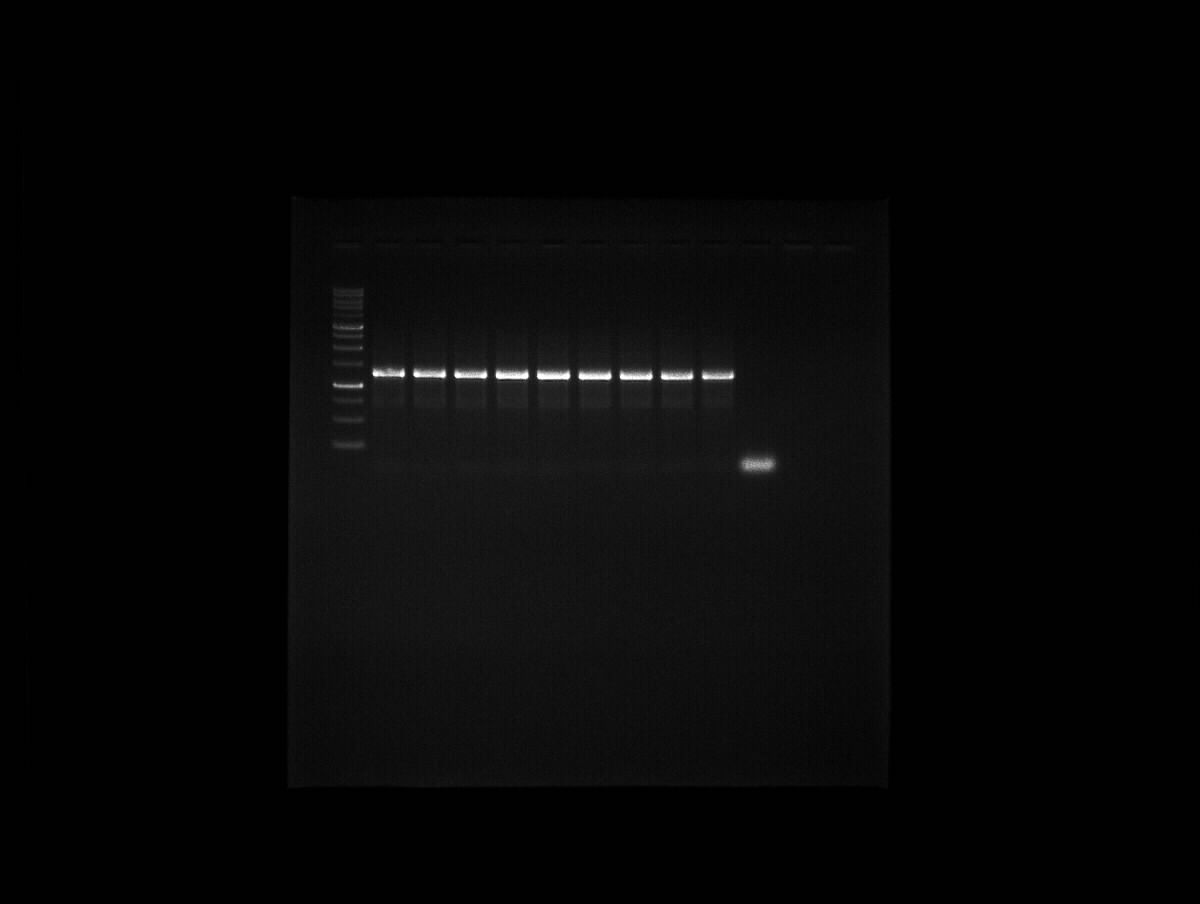


M L1 L2 L3 L4 L5 L6 L7 L8 L9 L10

~1.2 kb

**Supplementary figure S2.** Nested PCR assay results of phytoplasma DNA amplification from eggplant little leaf and phyllody plants with primer pair R16F2n/R16R2; M: 1Kb Ladder, Lane 1: BLLP-1, Lane 2: BLLP-2, Lane 3: BLLP-3, Lane 4: BLLP-4, Lane 5: BLLP-5, Lane 6: BLLP-6, Lane 7: BLLP-7, Lane 8: BLLP-8, Lane 9: BLLP-9 and Lane 10: Negative control


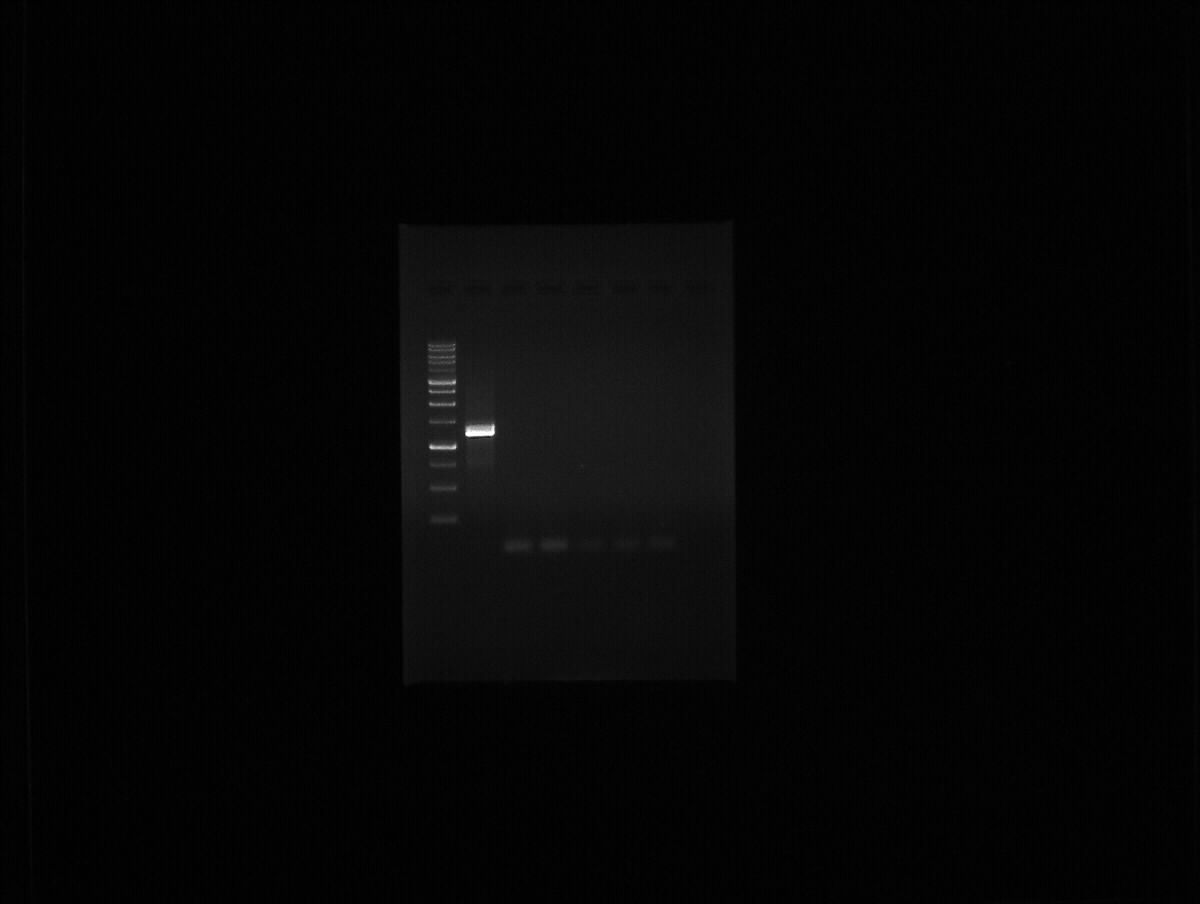


~1.2 kb

M L1 L2 L3 L4 L5 L6

**Supplementary figure S3.** Nested PCR assay results of phytoplasma DNA amplification from insect collected with primer pair R16F2n/R16R2; M: 1Kb ladder, Lane 1: *Hishimonus phycitis*, Lane 2: *Amrasca biguttula biguttula*, Lane 3: *Leucinodes arbonalis*, Lane 4: Henosepilachna vigintioctopunctata, Lane 5: *Bemisia tabaci* and Lane 6: Negative control
